# Role of Trientine in Hypertrophic Cardiomyopathy: A Review of Mechanistic Aspects

**DOI:** 10.3390/ph15091145

**Published:** 2022-09-14

**Authors:** Fitri Fareez Ramli, Syed Alhafiz Syed Hashim, Betty Raman, Masliza Mahmod, Yusof Kamisah

**Affiliations:** 1 Department of Pharmacology, Faculty of Medicine, Universiti Kebangsaan Malaysia, Kuala Lumpur 56000, Malaysia; 2 Clinical Psychopharmacology Research Unit, Department of Psychiatry Warneford Hospital, University of Oxford, Oxford OX3 7JX, UK; 3 Division of Cardiovascular Medicine, Radcliffe Department of Medicine, John Radcliffe Hospital, University of Oxford, Oxford OX3 9DU, UK

**Keywords:** cardiomyopathy, diabetes, hypertensive, TETA, triethylenetetramine

## Abstract

Abnormality in myocardial copper homeostasis is believed to contribute to the development of cardiomyopathy. Trientine, a copper-chelating drug used in the management of patients with Wilson’s disease, demonstrates beneficial effects in patients with hypertrophic cardiomyopathy. This review aims to present the updated development of the roles of trientine in hypertrophic cardiomyopathy. The drug has been demonstrated in animal studies to restore myocardial intracellular copper content. However, its mechanisms for improving the medical condition remain unclear. Thus, comprehending its mechanistic aspects in cardiomyopathy is crucial and could help to expedite future research.

## 1. Introduction

Heart failure affects more than 64 million people globally [1], with its prevalence ranging from 0.12 to 6.70% [2]. It is a complication of other diseases such as diabetes mellitus [3], ischemic heart disease, or long-standing hypertension [4], or primarily due to genetic and idiopathic conditions [5]. These conditions can give rise to the development of cardiomyopathy. Myocardial hypertrophy is the most common cause of heart failure [6], characterized by enlargement of the heart [3].

Myocardial hypertrophy occurs following remodeling of the heart, triggered by various stimuli. Remodeling causes myocardial structural, molecular, and cellular changes, leading to alterations in cardiac function, size, and shape [7]. Many pathological events, such as neurohormonal activation, cardiac volume overload, and pressure overload are thought to be centrally involved in the development of cardiac hypertrophy [7]. Deficiencies in micronutrients such as copper, zinc, and selenium may also play a role in the development of heart failure [8]. Abnormality in copper homeostasis manifested by a deficiency in myocardial copper may contribute to the pathogenesis of cardiac hypertrophy [9]. Patients with ischemic heart disease tend to have a lower cardiac copper content [10], possibly due to increased myocardial copper efflux [11] and reduced activity in certain copper-dependent enzymes [10]. Animals fed a diet deficient in copper had significantly heavier hearts [12], owing to alterations in heart biochemical properties and morphology [13]. Similar observations were also noted in pressure-overloadinduced hypertrophied hearts in rats [14,15].

The exact mechanism of how copper deficiency induces cardiac hypertrophy is not well understood but may be partly attributable to dietary and hereditary etiologies. Genetic polymorphism in copper-transporting ATPase has been postulated to cause pathological cardiac changes, as observed in Menkes’ disease, an X-linked genetic defect affecting energy-dependent copper transporters [16]. Copper plays an important role in many cellular processes such as antioxidant activity and mitochondrial respiration [17]. Dietary supplementation of copper reversed cardiac hypertrophy in rats [18].

Cardiac hypertrophy and heart failure are managed pharmacologically using angiotensin-converting enzyme inhibitors, angiotensin receptor blockers, calcium channel blockers [19], β-blockers, or angiotensin receptor–neprilysin inhibitors [20,21,22]. Due to the discovery of copper-deficiency-induced cardiac hypertrophy, clinical trials have been conducted to investigate the effects of trientine, a copper chelator, on cardiac hypertrophy or hypertrophic cardiomyopathy [23,24].

**Figure 1:**
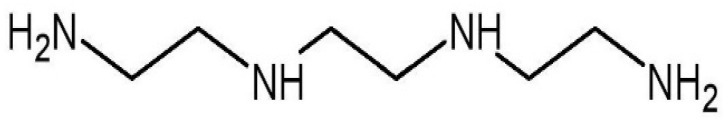
Molecular structure of trientine.

Trientine, also known as triethylenetetramine (TETA) dihydrochloride (Figure 1), is an orphan drug [25]. It is an organic compound with a molecular formula of C_6_H_18_N_4_, which was originally approved in 1985 by the U.S. Food and Drug Administration as second-line therapy for patients with Wilson’s disease who cannot tolerate penicillamine [26,27]. Wilson’s disease is a genetic condition that arises from copper accumulation in the body, particularly the liver. This could lead to liver cirrhosis and degenerative neurological conditions which could be fatal [28]. Trientine chelates hepatic excess copper and increases urinary copper excretion, leading to hepatic improvement in the majority of patients with the disease. Other copper modulators are dimercaprol and zinc salts (acetate, sulfate, and gluconate) [27].

The role of trientine in cardiomyopathy has gained much interest, although its mechanistic action is poorly understood. This review aims to provide an overview of the molecular aspects of the drug’s effects on cardiac hypertrophy, a precursor for heart failure. It can enhance our understanding of its beneficial effects and encourage further research in the field.

## 2. Role of Trientine in Cardiac Copper Regulation

Copper is required for the normal function and structure of the heart. It is involved in various cellular metabolic activities such as iron and zinc uptake, as well as oxyradical scavenging [17]. It enters cells via plasma membrane copper transporter-1 (CTR-1) and copper transporter-2 (CTR-2) [29]. Once inside the cells, the ion is delivered to its targets by various chaperones [17]. However, in excess, it may be detrimental due to the generation of reactive oxygen species (ROS). Consequently, its uptake, distribution, and elimination are tightly governed to maintain cellular homeostasis [29,30].

The serum copper level is higher in patients with cardiomyopathy compared with that of normal individuals [31]. It is postulated that increased efflux of copper from the myocardial cells leads to a high level of serum copper [32]. To confirm this, many animal model experiments using various cardiomyopathy models such as pressure-overload-induced cardiac hypertrophy [14] or diabetic cardiomyopathy [33] have been conducted. Findings from the studies confirmed the depletion of myocardial copper content measured in left ventricles [14,15,33]. The depleted content could not be replenished even in the presence of higher circulatory copper levels [11]—a phenomenon which remains elusive.

Copper exists in two valencies: Cu(I) and Cu(II). Cu(I) is mainly present intracellularly and contributes 95% of total body copper, and the remainder exists as Cu(II) in the extracellular space [34]. Its transport intracellularly is governed by various chaperones which will be discussed in the subsequent subtopics. Trientine selectively forms a complex with Cu(II), not Cu(I) [35], and serves as a copper chaperone to transport the ion to other copper-binding molecules in copper-depleted cardiomyocytes [14]. However, the mechanism is still unclear. Cu(II)-trientine enters cells as an intact complex via active transport [11]. CTR-1 expression is the major copper transporter in the heart. However, trientine does not affect myocardial CTR-1 expression [14]. Even in diabetic myocardium, it does not restore the diminished CTR-1 protein expression [34]. In cardiomyocytes transfected with CTR-1 gene silencing using siRNA, trientine-facilitated copper uptake into cardiomyocytes was unaffected, observed by increased copper accumulation in the cells. This was different from the cells that were exposed to Cu(II) chloride only, which demonstrated a decline in cellular copper content [11], confirming that the influx of copper into cells by trientine is CTR-1-independent. Studies investigating the effects of trientine on CTR-2 reported inconsistent findings (Table 1). Trientine exhibited elevated CTR-2 expression in a few studies [15,34] but no effect in another study [14]. This suggests that CTR-2 may partly contribute to copper transportation into cardiomyocytes. CTR-2 expression in the heart is determined by the cellular copper status, which is reduced in copper deficiency [36]. Therefore, trientine may upregulate CTR-2 expression indirectly due to the accumulated copper in cardiomyocytes. Another transporter that may be involved in copper transport is chloride channel CLC17 [37], and the effects of trientine on the transporter should be investigated. More studies need to be conducted to better understand the mechanisms of copper uptake by the drug.

Further studies in animals demonstrated that trientine restored myocardial copper content in cardiomyopathy models [14,34,35] (Table 1). It was previously revealed that trientine at a low dose (21.9 mg/kg, orally twice daily for 6 weeks) replenished deprived copper in the heart, but a relatively high dose (87.6 mg/kg, orally twice daily), an equivalent dose commonly used for the treatment of Wilson’s disease, failed to reload the loss of myocardial copper content in rats with pressure-overload-induced cardiac hypertrophy. Moreover, the high dose of trientine decreased copper content in the heart of normal rats, whereas the same phenomenon was not observed with the low dose [14]. The findings suggest that trientine at a relatively high dose of more than 175 mg/kg/day promotes the removal of copper from the heart. The protective effect of trientine on the myocardial copper content agreed with other animal studies that adopted a trientine dosage of approximately 100 mg/kg/day or less [15,33,34,35,38,39].

Administration of trientine exhibited protection against left ventricular hypertrophy in diabetic patients [40]. Clinical trials reported that serum copper was unaltered by trientine therapy after six [23] or twelve months [40] in patients with hypertrophic cardiomyopathy. Yet, the 24 h urinary copper level was significantly elevated in the patients, indicating increased excretion of excess copper (Table 1). However, a small significant rise in serum ceruloplasmin, the main copper-bearing protein in the blood, occurred in the patients, indicating increased cellular uptake of copper [23].

Despite the effects of trientine on myocardial copper content, it has no significant effects on plasma copper levels in rats, even though it reduces renal copper content and facilitates urinary excretion of excessive copper in the blood [14,35,39]. In other words, trientine therapy is not likely to cause systemic copper deficiency. A similar insignificant effect on plasma copper was also observed in patients with left ventricular hypertrophy who were receiving trientine. The effectiveness of the therapy was monitored by a decrease in left ventricular mass [40].

Regardless of its beneficial effects on diabetes-induced copper depletion in the heart [34,39], trientine has no significant effect on blood glucose levels in diabetic rats [25,34,39,41,42]. How it offers cardioprotection without affecting blood glucose is not understood. More mechanistic studies in experimental animals should be conducted to elucidate the mechanism.

**Table 1:** The effects of trientine on cardiac copper regulation in animals and related blood parameters in animals and patients with hypertrophy.

Study	Type of Model/Subjects	Trientine (TETA) (Dose and Duration)	Findings	Reference
Animal	STZ-induced diabetic cardiomyopathy in rats	20 mg/day in drinking water for 8 weeks (post-treatment) (~68 mg/kg/day)	↑ cardiac copper content ↔ CTR-1 mRNA and protein ↑ CTR-2 mRNA and protein	[34]
Animal	STZ-induced diabetic cardiomyopathy in rats	20 mg/day in drinking water for 8 weeks (post-treatment) (~68 mg/kg/day)	↑ cardiac copper content	[38]
Animal	Transverse aortic constriction- induced cardiac hypertrophy in rats	21.9 and 87.6 mg/kg twice daily orally for 6 weeks	TETA (21.9 mg/kg/day): ↑ LV copper content ↑ urinary copper ↓ renal copper content ↔ plasma copper level ↔ CTR-1 and CTR-2 protein TETA (87.6 mg/kg/day): ↓ LV and renal content ↑ urinary copper ↓ renal copper content ↔ plasma copper level ↔ CTR-1 and CTR-2 protein	[14]
Animal	Ascending aortic constriction-induced cardiac hypertrophy in rats	21.9 mg/kg twice daily orally for 6 weeks	↑ cardiac copper ↔ CTR-1 mRNA and protein ↑ CTR-2 mRNA and protein	[15]
Animal	STZ-induced diabetic cardiomyopathy in rats	(a) Intravenous infusion, 60 s once hourly in increasing doses (0.1, 1.0, 10, and 100 mg/kg) (b) 8–11 mg/day in drinking water for 7 weeks (post-treatment)	↑ Copper urinary excretion ↑ Cardiac total copper	[39]
Animal	STZ-induced diabetic cardiomyopathy in rats	30 mg/day in drinking water for 8 weeks (post-treatment)	↑ LV total copper	[33]
Human	Type 2 diabetic patients with LVH (*n* = 15)	600 mg twice daily orally for 12 months	↔ plasma copper ↑ 24 h urinary copper	[40]
Human	Patients with hypertrophic cardiomyopathy (*n* = 20)	300 mg twice daily orally, increased after 1 week to 600 mg twice daily if tolerated for 6 months	↔ serum copper	[23]

CTR-1, copper transporter 1; CTR-2, copper transporter 2; LV, left ventricle; LVH, left ventricular hypertrophy; STZ, streptozotocin; ↑, significant increase; ↓, significant decrease; ↔, no difference.

## 3. Effects of Trientine on Mitochondrial Function and Biogenesis

Copper is an essential trace element that serves as a co-factor in various enzyme activities involved in mitochondrial ATP production [8,43]. Copper is delivered to its target molecules in various organelles by chaperones [8]. Upon entering cells, copper is fetched by cytosolic copper chaperones. Cytosolic soluble cytochrome c oxidase (Cco) copper chaperones 17 (Cox17) and 11 (Cox11) are chaperones that shuttle copper to other chaperones such as mitochondrial-inner-membrane-bound Cco-assembly proteins—Sco1 and Sco2—which then deliver the copper to Cco in the mitochondria [37,44]. Cco is a copper-dependent enzyme that is actively involved in oxidative phosphorylation in the electron transport chain [44]. In cardiomyopathy, its activity is reduced [38,45].

**Figure 2:**
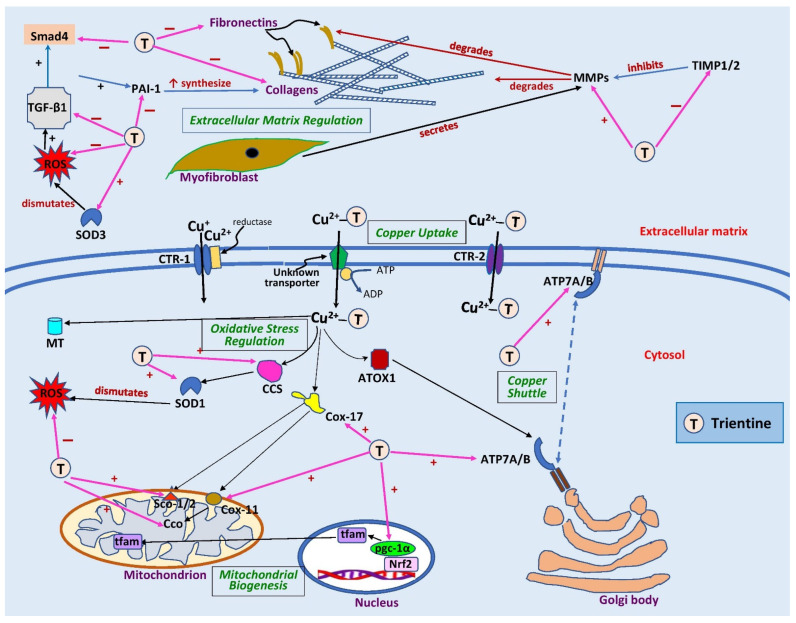
Sites of action of trientine in cardiac remodeling. Extracellularly, trientine reduces interstitial fibrosis by inhibiting tissue inhibitor of metalloproteinase 1/2 (TIMP1/2), leading to an increase in matrix metalloproteinases (MMPs). This, in turn, augments collagen degradation. Inhibitory effects of trientine on transforming growth factor-β1 (TGF-β1), plasminogen activator inhibitor-1 (PAI-1), and small mothers against decapentaplegic (Smad4) lead to decreases in expression of matrix proteins (collagen and fibronectin). The complex of trientine-Cu(II) is brought into cells by an unknown transporter and partly by copper transporter-2 (CTR-2). The drug increases the expression of superoxide dismutase-1 (SOD1) and -3 (SOD3) as well as copper chaperone for superoxide dismutase-1 (CCS), leading to a decrease in reactive oxygen species (ROS) formation. Trientine also elevates the expression of cytosolic soluble cytochrome c oxidase (Cco) copper chaperones 11 (Cox11) and 17 (Cox17), cytochrome c oxidase assembly protein-1/2 (Sco1/2), and peroxisome proliferator-activated receptor coactivator-1α (pgc-1α), which improves mitochondrial biogenesis and function. It also augments the expression of ATPase copper-transporting α (ATP7A) but has no effect on ATPase copper-transporting β (ATP7B) or antioxidant 1 copper chaperone (ATOX1), which are responsible for shuttling copper into trans-Golgi network or secretory vesicles. CTR-1, copper transporter 1; MT, metallothionein; nrf2, nuclear factor erythroid 2-related factor 2; tfam, mitochondrial transcription factor A; ↑, significant increase; +, stimulates; −, inhibits. Blank arrows demonstrate subsequent events; brown arrow indicates degradation, dashed blue arrow displays translocation of copper, and pink arrows indicate the sites of action of trientine.

Only two studies have investigated the effects of trientine on myocardial mitochondrial function and biogenesis. Zhang et al. [38] demonstrated that myocardial copper replenishment by trientine in the copper-deficient myocytes of diabetic rats restored normal copper trafficking between the cytoplasm and mitochondria, as evidenced by an increase in gene expressions of copper chaperones Cox17, Cox11, and Sco1 (Table 2) (Figure 2). The drug increased Cco subunits I (mt-coI) and II (mt-coII) gene expression but did not affect subunit III (mt-coIII). However, trientine did not alter the protein expression of the subunits [38]. Future studies should be conducted to investigate the effects of the drug on the mitochondrial membrane potential in cardiomyopathy since Cco and Cox17 directly affect the potential. The mitochondrial membrane potential is a crucial entity in energy storage during oxidative phosphorylation. Disturbance in the potential generation could result in mitochondrial dysfunction [46].

Trientine also enhanced the expression of ATPase copper-transporting α (ATP7A) localization and its gene expression (Atp7a) in left ventricular diabetic rats [34]. ATP7A is a copper chaperone that delivers copper to ceruloplasmin in the Golgi bodies [37,44]. In terms of mitochondrial DNA synthesis, trientine did not influence mitochondrial transcription factor A (mt-tfam) or single-strand DNA-binding protein (mt-ssbp) gene expression, leading to no change in mitochondrial DNA content. However, it augmented the expression of peroxisome proliferator-activated receptor coactivator-1α (pgc-1α), a principal regulator of mitochondrial metabolism [38].

The heart has a high energy metabolism, hence it requires an energy depot for an immediate supply of ATP. Phosphocreatine represents energy storage for the rapid production of ATP. The former converts to creatine, releasing its phosphate that phosphorylates ADP, synthesizing ATP [47]. Hence, the ratio could serve as an indicator of cardiac high-energy phosphate metabolism [48]. This can be measured by using phosphorus magnetic resonance spectroscopy [49]. In patients with abnormalities in cardiac metabolism, the myocardial phosphocreatine/ATP ratio declines, and this has been shown to be prognostically relevant in models of heart failure [48]. Only one clinical trial in phase 2a has explored the effects of trientine on cardiac energetics in 20 patients with hypertrophic cardiomyopathy [23]. They found that trientine therapy at 600 mg twice daily for six months produced a non-significant 10% rise in the left ventricular phosphocreatine/ATP ratio, suggesting the potential of trientine to restore mitochondrial function and energetics in cardiomyopathy.

Collectively, trientine therapy can reverse cardiomyocyte-defective copper metabolism in cardiac hypertrophy or cardiomyopathy. This could improve mitochondrial function and bioenergetics, driving amelioration of heart structure and function. More studies on animals investigating this aspect should be conducted. Trientine may affect sirtuin-3, which is crucially involved in mitochondrial metabolism. It warrants further investigation to appreciate its role in mitochondrial function and biogenesis.

## 4. Effects of Trientine on Cardiac Function

Disturbances in myocardial copper homeostasis affect mitochondrial function and then cardiac function. Only two studies have reported the effects of trientine on cardiac functions in patients with cardiomyopathy [23,40]. Reid et al. [23] reported that trientine therapy (600 mg twice daily) for six months improved left ventricular function, evidenced by improved global longitudinal strain (GLS) and peak mitral annular systolic (S′) velocity—sensitive markers of left ventricular function—in patients with hypertrophic cardiomyopathy (Table 3). The therapy also ameliorated left atrial impairment, accompanied by decreased left atrial end-systolic volume and elevated total atrial strain [23]. The findings from the study suggest a potential therapeutic effect of trientine in patients with cardiomyopathy, with improvements in left ventricular and atrial function. However, left ventricular ejection fraction (LVEF)—a less sensitive marker than GLS at detecting left ventricular systolic dysfunction [50]—and left atrial ejection fraction, as well as left ventricular end-systolic (LVESV) and end-diastolic (LVEDV) volume, were unaltered by the therapy. Prolonging the duration of therapy to 12 months did not seem to produce any beneficial outcomes on LVEF, LVESV, or LVEDV in patients with diabetic cardiomyopathy [40].

In animals, trientine treatment at approximately 34–68 mg/kg/day for 6–8 weeks increased cardiac output, maximal rate of fall (−dp/dt_max_) and rise (+dp/dt_max_) of left ventricular pressure, left ventricular fractional shortening (LVFS), and LVEF, as well as decreasing LVESV (Table 3) [15,25,33,34,39,41,42,51,52]. The parameters assess left ventricular systolic function [53]. It also improved left ventricular diastolic function, observed by a reduction in LVEDV and left ventricular end-diastolic pressure (LVEDP) [41,51]. However, the left atrium function was not explored in these studies. The improvements may be explained by the restoration of copper homeostasis, effective cellular respiration, and antioxidant defense mechanism, leading to structural improvement by trientine. Concurrent deterioration in structural and functional aspects of the heart in the copper depletion state indicates an essential role of copper in the physiological function of the heart and a beneficial role of trientine in reversing these changes. More studies investigating the effects of the drug on the left atrium should be carried out to shed more light on its potential actions.

Notably, trientine also impeded heart-rate-corrected QT interval (QTc) prolongation in rats with streptozotocin-induced diabetic cardiomyopathy [33]. The QT interval is normally prolonged in patients with diabetes, which can be associated with an increased risk of cardiovascular diseases such as stroke and ischemic heart disease [54]. This finding suggests that copper is an essential element that regulates QT interval.

Myocardial calcium homeostasis, which plays an important role in myocardial contractility, is disturbed in diabetic cardiomyopathy [55]. Physiologically, calcium released into the cytoplasm from the sarcoplasmic reticulum is returned to the sarcoplasmic reticulum by sarcoplasmic/endoplasmic reticulum calcium ATPase 2a (SERCA2a). The sodium–calcium exchanger (NCX) enhances the sodium–calcium exchange when the extracellular level of sodium is elevated [56]. Impaired myocardial contractility in diabetic rats was reversed by trientine therapy. This is possibly due to diminishing troponin I phosphorylation, which thereby elevates myofibrillar calcium sensitivity, resulting in a curb in cardiac remodeling [33]. Phosphorylation of troponin I promotes myocardial relaxation and declines myocardial contraction–relaxation cycle time [57]. However, trientine did not affect NCX and SERCA2a proteins [33]. More studies must be executed to further investigate the impacts of trientine on myocardial calcium regulation. The role of calcium-modulating proteins such as ryanodine receptor type 2 (RyR2) and FOK-binding protein/calstabin 2 (FKBP12.6), which are involved in calcium release, or phospholamban in calcium uptake [55,58] could be examined. Possible effects of trientine on calsequestrin 2 (CASQ2), which is responsible for calcium storage [59], should also be studied.

## 5. Effects of Trientine on Myocardial Oxidative Stress and Inflammation

Oxidative stress and inflammation are undeniably involved in cardiac damage and cardiomyopathy [60,61]. The elevation of these is observed in diabetes and prolonged pressure overload. These effects result in the development of various pathological changes such as mitochondrial structural and functional defects [38,62]. However, the role of trientine on oxidative stress and inflammation has not been extensively investigated. It has been demonstrated to improve both pathological conditions in the liver [63], but in the heart, only its effects on oxidative stress have been investigated [38]. In experimental diabetic cardiomyopathy models, trientine has been demonstrated to increase superoxide dismutase-1 (cytosolic SOD1) and superoxide dismutase-3 (SOD3, also known as extracellular SOD and EC-SOD) expression [34,38,42] (Table 4) (Figure 2). These enzymes are fundamentally important in the cells’ defense against oxidative stress [64]. Both isoenzymes contain copper and zinc [65,66]. Therefore, the increased activity of the enzymes following trientine therapy is an expected outcome and aligns with studies of diabetic rats, where copper chaperone for superoxide dismutase-1 (CCS) expression was increased [34,38]. Of note, trientine can also indirectly increase the enzyme synthesis, and thus its activity.

In diabetes, myocardial intracellular Cu(I) content is decreased, whereas chelatable Cu(II) in the myocardial extracellular space is approximately threefold higher [39]. Cu(II) can promote the production of ROS such as superoxide anion and hydroxyl radicals [65]. SOD is responsible for converting the superoxide anion to hydrogen peroxide before being detoxified by other antioxidant enzymes [67]. Trientine also likely elevates myocardial antioxidant capacity by diminishing Cu(II)-induced ROS, possibly via metal chelation. This could provide further protection to cardiomyocytes by mitigating fibrosis since oxidative stress and inflammation are known to induce fibrosis.

To maintain cellular copper homeostasis, metallothionein ensures excess intracellular copper is sequestered. Metallothionein is a copper-binding protein, and its synthesis is regulated by copper and zinc availability [44]. Trientine has no significant effect on myocardial metallothionein expression (Table 4); however, it decreases the protein polymerization. This suggests that the availability of redox-sensitive metallothionein is increased [34], conferring more protection against copper-induced toxicity. This phenomenon is consistent with the higher intracellular copper level induced by trientine therapy. The drug also does not influence the expression of human antioxidant protein 1 (ATOX1) [34], a chaperone that passes copper to ATPase, including ATPase copper-transporting α (ATP7A) and ATPase copper-transporting β (ATP7B). The ATPases then shuttle the copper to the trans-Golgi network or secretory vesicles [44]. In diabetic cardiomyopathy, the expression of ATP7B is impaired, whereas that of ATP7A is unaffected. Trientine increases ATP7A expression but does not affect ATP7B [34]. The increased expression of the former may be to compensate for the diminished function of the latter induced by diabetes.

More studies on experimental animals are needed to better understand the effects of trientine on myocardial oxidative stress and inflammation in cardiomyopathy. Trientine may modulate inflammatory signaling pathways such as nuclear factor kappa-light-chain-enhancer of activated B cells (NF-κB) and calcineurin–nuclear factor of activated T cells (NFAT) signaling pathways or nucleotide-binding domain leucine-rich repeat family pyrin domain containing receptor 3 (NLRP3) inflammasome. Nuclear factor erythroid 2-related factor 2/antioxidant responsive element (Nrf2/ARE) and Kelch-like ECH-associated protein 1 (Keap1) signaling pathways, which are involved in the regulation of oxidative stress [68], could also be studied.

## 6. Effects of Trientine on Extracellular Matrix Regulation and Interstitial Fibrosis

The cardiac extracellular matrix plays a crucial role in cardiac remodeling. Two main components in the matrix are collagen types I and III, with cardiac fibroblast being the main producer [69]. Dysregulation of the extracellular matrix precipitates myocardial interstitial fibrosis, a progressive pathological feature in cardiomyopathy [70] following an excessive accumulation of extracellular matrix proteins in the heart. These events lead to distortion and stiffness of cardiac structure [71]. Collagens are degraded by matrix metalloproteinases (MMPs), which are governed by tissue inhibitors of metalloproteinase (TIMP). This provides a balance between the degradation and synthesis of extracellular matrix protein [69]. Increased collagen content, particularly in the left ventricles, may perturb cardiac contractility and myofibrillar Ca^2+^ sensitivity, which in turn promotes cardiac dysfunction [14].

In rats, trientine treatment decreased left ventricular collagen types I and III expression in streptozotocin-induced diabetic cardiomyopathy [33,39,42] and pressure-overload-induced cardiac hypertrophy [14,15], reflected by a decrease in hydroxyproline content—a major component in collagen—and an increase in MMP-2 protein [15] (Table 5). The antifibrotic effect of trientine was further strengthened by a diminished expression of TIMP-1 and TIMP-2 [15] (Figure 2). Replenished myocardial copper by trientine has an indirect effect on MMP-2 activity. MMP-2 is regulated by hypoxia-inducible factor-1 (HIF-1), which requires copper for its transcriptional activity [72]. Moreover, MMP-2 activity is zinc-dependent: zinc uptake is dependent on copper status [73]. MMP-2 activity is also inversely affected by lysyl oxidase, a copper-dependent enzyme involved in collagen–elastin crosslinking [74]. Thus, MMP-2 expression is indirectly regulated by trientine through its increment of cellular copper uptake.

Trientine therapy (600 mg twice daily) for six months in patients with hypertrophic cardiomyopathy improved left ventricular function, evidenced by reduced extracellular matrix volume. However, no effect on extracellular volume fraction was noted, measured via cardiac magnetic resonance imaging [23]. This indicates that the therapy decreased the expansion of the extracellular matrix, which could be due to the buildup of insoluble proteins in the left ventricle [75]. Negative finding may imply that the sample size of the study could have been too small to see a significant effect. It is also possible that other mechanisms are at play in humans which need further studies for a confirmation.

Transforming growth factor-β1 (TGF-β1) is one of the regulators involved in the pathogenesis of cardiac fibrosis. It is activated after cardiac injury by stimulating collagen synthesis [76], mediated by the small mothers against decapentaplegic (Smad) protein [77]. TGF- β1 also promotes plasminogen activator inhibitor-1 (PAI-1) via Smad to stimulate tissue fibrosis [78]. Only a single study has investigated the effects of trientine on the TGF-β1/Smad4 signaling pathway. The drug decreased left ventricular TGF-β1, PAI-1, and Smad4 gene expression in diabetic cardiomyopathy rats, associated with reductions in expression of collagen and fibronectin-1—a matrix protein [42] (Figure 2). An elevated extracellular Cu(II) level enhances the buildup of advanced glycated end-products in collagen, which then promotes collagen deposition triggered by TGF-β1 [42]. Therefore, the reduction in Cu(II) by trientine likely prevents the buildup of advanced glycated end-products, leading to a decrease in TGF-β1 expression.

Taken together, trientine ameliorates myocardial fibrosis via the Smad signaling pathway, reducing the collagen content in the myocardium and its associated fibrotic factors. Unfortunately, few in-depth studies have been conducted to investigate the impacts of trientine therapy on myocardial fibrosis. Many aspects such as frizzled-related protein 1 (sFRP-1)/Wnt/β-catenin, phosphatidylinositol 3-kinase/protein kinase B/glycogen synthase kinase-3β (PI3K/Akt/GSK-3β), and hypoxia-induced mitogenic factor-interleukin 6 (HIMF-IL-6) signaling pathways, which have a significant role in myocardial fibrosis regulation [22], are yet to be studied. Trientine may modulate these pathways. The role of trientine on other collagen-degrading proteinases such as cathepsin L in the heart should also be explored.

## 7. Effects of Trientine on Cardiac Structure

Myocardial copper deficiency results in cardiac hypertrophy [14,15]. As mentioned earlier, copper deficiency diminishes Cco activity which then impairs mitochondrial function. This drives compensatory increases in mitochondrial biogenesis and size, leading to hypertrophy [79]. Subsequently, defects in mitochondrial structures characterized by elevated volume density, fragmentation, vacuolation, and reduction or disappearance of mitochondrial cristae occur [9]. Treatment with trientine significantly reduced the ratio of heart weight to body weight in rats with diabetic cardiomyopathy and pressure-overload-induced hypertrophy [34,39,42,51] (Table 6). It reduced the size of cardiomyocytes and improved diastolic left ventricular wall thickness in the rats.

The improvement by trientine in structural parameters of diabetic cardiomyopathy in animal studies was confirmed by positive findings in a randomized clinical controlled trial. Cooper et al. [40] reported a significant reduction in left ventricular hypertrophy mass indexed to body surface area at six and twelve months in the trientine arm (600 mg twice daily for 12 months) in patients with diabetic cardiomyopathy compared to placebo. However, Reid et al. [23] noted a non-significant effect of trientine at a similar dose after six months on the same parameter in patients with hypertrophic cardiomyopathy. One possible explanation for the discrepancy in results between the two studies is the shorter duration of trientine therapy and the difference in the types of patients compared with the former study [40].

A transient significant increase in 24 h urinary copper excretion at four months after trientine therapy was observed in diabetic cardiomyopathy patients, thereafter, gradually declining after eight months [40]. Increased urinary copper excretion by trientine removes excess copper from the myocardial extracellular space [39], resulting in attenuated ROS generation and finally reducing fibrosis. This partly ameliorates the cardiac structure and eventually restores cardiac function. Moreover, the formation of stabilized collagen fibril crosslinking requires lysyl oxidase [81]. The enzyme activity is diminished in copper-deficient bovine hearts [82]. Attenuation of the enzyme activity impairs collagen crosslinking that wanes the myocardium structure, resulting in raised myocardial workload to pump blood [9]. Normalized myocardial intracellular copper by trientine can be postulated to improve cardiac structure and restore its function. However, El Hajj et al. [83] reported that the expression of lysyl oxidase was positively correlated with collagen deposition in the heart. The increased expression of the enzyme was associated with a decrease in cardiac function. How trientine affects the enzyme activity that leads to cardiac structure improvement is yet to be determined.

Improved copper trafficking across and within myocytes could also contribute to the cardioprotective effects of trientine on cardiac structure [14,15,38], owing to improved mitochondrial function and biogenesis [34,38]. More large-scale clinical studies must be conducted to determine a conclusive effect of trientine on cardiomyopathy.

## 8. Conclusions and Directions for Future Studies

Trientine has great potential for the treatment of hypertrophic cardiomyopathy given its ability to improve the copper content and structural and functional aspects of the heart. Although the findings were mixed, the weight of evidence points to net benefit from trientine on cardiac structure and function. However, many perspectives of cardiac remodeling in cardiomyopathy must be explored. To date, no studies have investigated the potential effects of trientine on hypertrophic factors such as zinc finger transcription factor GATA-binding protein 4 (GATA4) or CCAAT-enhancer-binding proteins (C/EBPs). Apoptosis also plays a major role in cardiomyopathy. Elucidation of the effects of trientine on signaling pathways of survivor-activating factor enhancement (SAFE) implicated in cardiomyocyte survival [84] and 5′ adenosine monophosphate-activated protein kinase/mammalian target of rapamycin/ribosomal protein S6 kinase beta-1 (AMPK/mTOR/p70S6K) associated with autophagy [85] could help further understanding. Figure 2 depicts the sites of action of trientine in cardiac remodeling.

To the best of our knowledge, findings from only two clinical trials with a small sample size that explicate the effects of trientine on cardiomyopathy have been published. Large-scale randomized clinical trials should be performed to confirm its beneficial effects for this clinical use. Currently, the large-scale multicenter, double-blind randomized placebo-controlled Trientine in Hypertrophic Cardiomyopathy (TEMPEST) phase 2 clinical trial by a research group from the University of Oxford, United Kingdom, is ongoing. The trial intends to evaluate the impacts of trientine on myocardial energetics in patients with hypertrophic cardiomyopathy. The outcomes may confirm the clinical utilization of trientine for the treatment of hypertrophic cardiomyopathy.

## Figures and Tables

**Table 2:** Effects of trientine on copper chaperones involved in cardiac mitochondrial function and biogenesis in human and animal studies.

Study	Type of Model/Subjects	Trientine (Dose and Duration)	Findings	Reference
Animal	STZ-induced diabetic cardiomyopathy in rats	20 mg/day in drinking water for 8 weeks (post-treatment) (~68 mg/kg/day)	↑ ATP7A ↑ Atp7a mRNA	[34]
Animal	STZ-induced diabetic cardiomyopathy in rats	20 mg/day in drinking water for 8 weeks (post-treatment) (~68 mg/kg/day)	↑ mt and cyto cox17 mRNA and protein ↑ mt and cyto cox11 protein ↑ mt Sco1 protein in LV ↑ mitochondrial Cco activity ↑ mt-coI mRNA ↑ mt-coII mRNA ↔ mt-coIII mRNA ↔ mt-coI protein ↔ mt-coII protein ↔ mt-coIII protein ↔ mt-DNA content ↔ mt-tfam mRNA ↔ mt-ssbp mRNA ↑ pgc-1α mRNA	[38]
Human	Patients with hypertrophic cardiomyopathy (*n* = 20)	300 mg twice daily orally, increased after 1 week to 600 mg twice daily if tolerated for 6 months	↔ PCr/ATP ratio	[23]

ATP7A or Atp7a, ATPase copper-transporting α; cox11, cytosolic soluble cytochrome c oxidase (Cco) copper chaperone 11; cox17, Cco copper chaperone 17; cyto, ctytosol; LV, left ventricular; mt, mitochondria; mt-coI, mitochondrial cytosolic soluble cytochrome c oxidase (Cco) subunit I; mt-coII, mitochondrial Cco subunit II; mt-coIII, mitochondrial Cco subunit III; mt-tfam, mitochondrial transcription factor A; mt-ssbp, mitochondrial single-strand DNA-binding protein; PCr, phosphocreatine; pgc-1α, peroxisome proliferator-activated receptor coactivator-1α; Sco1, cytochrome c oxidase assembly protein-1; STZ, streptozotocin; ↑, significant increase; ↓, significant decrease; ↔, no difference.

**Table 3:** Effects of trientine on cardiac function in human and animal studies.

Study	Type of Model/Subjects	Trientine (TETA) (Dose and Duration)	Findings	Reference
Human	Type 2 diabetic patients with LVH (*n* = 15)	600 mg twice daily orally for 12 months	↔ LVEDV ↔ LVESV ↔ LVEF ↔ E/E′	[40]
Human	Patients with hypertrophic cardiomyopathy (*n* = 20)	300 mg twice daily orally, increased after 1 week to 600 mg daily if tolerated for 6 months	↓ GLS ↔ LVEDV ↔ LVESV ↔ SV ↔ LVEF ↔ E/A ↔ HR ↔ Native septal T1 ↓ LAESV ↔ LAEDV ↔ LAEF ↓ Total atrial strain ↑ Mean S′ velocity	[23]
Animal	Transverse aortic constriction- induced cardiac hypertrophy in rats	21.9 and 87.6 mg/kg twice daily orally for 6 weeks	Both doses: ↔ LVESV ↔ LVEF	[14]
Animal	Ascending aortic constriction-induced cardiac hypertrophy in rats	21.9 mg/kg twice daily orally for 6 weeks	↑ LVEF ↑ LVFS	[15]
Animal	STZ-induced diabetic cardiomyopathy in rats	8–11 mg/day in drinking water for 7 weeks (post-treatment)	↑ CO ↑ LV +dp/dt_max_ ↑ LV −dp/dt_max_	[39]
Animal	STZ-induced diabetic cardiomyopathy in rats	20 mg/day in drinking water for 8 weeks (post-treatment)	↑ LV −dp/dt_max_	[42]
Animal	STZ-induced diabetic cardiomyopathy in rats	20 mg/day in drinking water for 8 weeks (post-treatment)	↑ CO	[25]
Animal	STZ-induced diabetic cardiomyopathy in rats	20 mg/day in drinking water for 8 weeks (post-treatment)	↑ HR ↑ CO ↑ LVEF ↔ LVEDV ↓ LVESV ↑ SV	[51]
Anima	STZ-induced diabetic cardiomyopathy in rats	30 mg/day in drinking water for 7 weeks (preventive)	Preventive: ↔ HR ↓ QTc interval Post-treatment: ↑ LV +dp/dt_max_ ↑ peak stress	[33]
Animal	STZ-induced diabetic cardiomyopathy in rats	20 mg/day in drinking water for 8 weeks (post-treatment)	↑ LV +dp/dt_max_ ↑ LV +dp/dt_max_ ↑ CO	[34]
Animal	STZ-induced diabetic cardiomyopathy in rats	20 mg/day in drinking water for 8 weeks (post-treatment) (~68 mg/kg/d)	↑ CO ↑ LV +dp/dt_max_ ↑ LV −dp/dt_max_	[39]
Animal	Zucker diabetic cardiomyopathy in rats	1 g/L in drinking water for 22 weeks (post-treatment)	↔ SV ↓ LVEDP ↓ LVEDV ↓ LVESV ↓ LV volume at +dp/dt_max_ ↓ LV volume at −dp/dt_max_	[41]
Animal	STZ-induced diabetic cardiomyopathy in rats	10 mg/day in the drinkingwater for 6 weeks (post-treatment)	↑ CO	[52]

CO, cardiac output; −dp/dt_max_, maximal rate of fall of left ventricular pressure; +dp/dt_max_, maximal rate of rise in left ventricular pressure; E/A, ratio of the early to the late peak diastolic transmitral flow velocity; E/E′, transmitral flow velocity/mitral annular velocity; GLS, global longitudinal strain; HR, heart rate; LAEDV, left atrial end-diastolic volume; LAEF, left atrial ejection fraction; LAESV, left atrial end-systolic volume; LV, left ventricle; LVEDP, left ventricular end-diastolic pressure; LVEDV, left ventricular end-diastolic volume; LVEF, left ventricular ejection fraction; LVFS, left ventricular fractional shortening; LVESV, left ventricular end-systolic volume; QTc, heart-rate-corrected QT; S′, peak mitral annular systolic; STZ, streptozotocin; SV, stroke volume; ↑, significant increase; ↓, significant decrease; ↔, no difference.

**Table 4:** Effects of trientine on myocardial oxidative stress and inflammation in animal studies.

Type of Model/Subjects	Trientine (Dose and Duration)	Findings	Reference
STZ-induced diabetic cardiomyopathy in rats	20 mg/day in drinking water for 8 weeks (post-treatment) (~68 mg/kg/day)	↑ mtSod1 activity ↑ mtSod1 protein ↑ mtCcs protein	[38]
STZ-induced diabetic cardiomyopathy in rats	20 mg/day in drinking water for 8 weeks (post-treatment) (~68 mg/kg/day)	↑ LV SOD3 mRNA	[42]
STZ-induced diabetic cardiomyopathy in rats	20 mg/day in drinking water for 8 weeks (post-treatment)	↑ Total antioxidant potential ↔ TNF-α	[25]
STZ-induced diabetic cardiomyopathy in rats	20 mg/day in drinking water for 8 weeks (post-treatment)	↑ SOD1 activity ↔ SOD1 protein ↑ Ccs protein ↑CCS ↔ ATOX1 ↑ ATP7A ↔ ATP7B ↔ Mt1 mRNA ↔ Mt2 mRNA ↓ MT (70 kD) protein ↑ MT (45 kD) protein ↑ MT (30 kD) protein	[34]

ATOX1, antioxidant 1 copper chaperone; ATP7A, ATPase copper-transporting α; ATP7B, ATPase copper-transporting β; Ccs or CCS, copper chaperone for superoxide dismutase-1; LV, left ventricle; MT, metallothionein; Mt1, metallothionein-1; Mt2, metallothionein-2; mtCcs, mitochondrial copper chaperone for superoxide dismutase-1; mtSOD1, mitochondrial superoxide dismutase-1; SOD1, superoxide dismutase-1; SOD3, superoxide dismutase-3; STZ, streptozotocin; TNF-α, tumor necrosis factor α; ↑, significant increase; ↓, significant decrease; ↔, no difference.

**Table 5:** Effects of trientine on myocardial interstitial fibrosis and extracellular matrix in human and animal studies.

Study	Type of Model/Subjects	Trientine (TETA) (Dose and Duration)	Findings	Reference
Animal	STZ-induced diabetic cardiomyopathy in rats	8–11 mg/day in drinking water for 7 weeks (post-treatment)	↓ LV collagen I ↓ LV collagen III	[39]
Animal	STZ-induced diabetic cardiomyopathy in rats	20 mg/day in drinking water for 8 weeks (post-treatment)	↓ LV collagen I, III, and IV mRNA ↓ LV fibronectin-I mRNA ↓ LV PAI-1 mRNA ↓ LV TGF-β1 mRNA ↓ LV Smad4 mRNA	[42]
Animal	Transverse aortic constriction- induced cardiac hypertrophy in rats	21.9 and 87.6 mg/kg twice daily orally for 6 weeks	TETA (21.9 mg/kg/day): ↓ cardiac collagen I TETA (87.6 mg/kg/day): ↔ cardiac collagen I	[14]
Animal	STZ-induced diabetic cardiomyopathy in rats	30 mg/day in drinking water for 8 weeks (post-treatment)	↓ LV collagen I ↔ LV collagen III	[33]
Animal	STZ-induced diabetic cardiomyopathy in rats	20 mg/day in drinking water for 8 weeks (post-treatment)	↓ TGF-β1	[25]
Animal	Ascending aortic constriction-induced cardiac hypertrophy in rats	21.9 mg/kg twice daily orally for 6 weeks	↓ cardiac collagen volume fraction ↓ cardiac hydroxyproline ↔ cardiac collagen I ↓ cardiac collagen III ↔ cardiac active MMP-9 ↓ cardiac MMP-9 mRNA ↑ cardiac active MMP-2 ↔ cardiac MMP-2 mRNA ↓ cardiac TIMP-1 mRNA ↓ cardiac TIMP-2 mRNA	[15]
Human	Patients with hypertrophic cardiomyopathy (*n* = 20)	300 mg twice daily orally, increased after 1 week to 600 mg twice daily if tolerated for 6 months	↔ ECV fraction ↓ ECM volume	[23]

ECM, extracellular matrix; ECV, extracellular volume; MMP, matrix metalloproteinases; LV, left ventricle; PAI-1, plasminogen activator inhibitor-1; Smad4, small mothers against decapentaplegic; STZ, streptozotocin; TGF-β1 transforming growth factor-β1; TIMP, tissue inhibitor of metalloproteinase; ↑, significant increase; ↓, significant decrease; ↔, no difference.

**Table 6:** Effects trientine on cardiac structure/hypertrophy in human and animal studies.

Study	Type of Model/Subjects	Trientine (TETA) (Dose and Duration)	Findings	Reference
Animal	STZ-induced diabetic cardiomyopathy in rats	20 mg/day in drinking water for 8 weeks (post-treatment) (~68 mg/kg/day)	↓ HW/BW	[38]
Animal	STZ-induced diabetic cardiomyopathy in rats	8–11 mg/day in drinking water for 7 weeks (post-treatment)	↓ HW/BW	[39]
Animal	STZ-induced diabetic cardiomyopathy in rats	20 mg/day in drinking water for 8 weeks (post-treatment)	↓ HW/BW	[42]
Animal	Ascending aortic constriction-induced cardiac hypertrophy in rats	21.9 mg/kg twice daily orally for 6 weeks	↓ cardiomyocyte size ↔ cardiac vimentin	[15]
Animal	STZ-induced diabetic cardiomyopathy in rats	20 mg/day in drinking water for 8 weeks (post-treatment)	↔ HW/BW	[25]
Animal	Transverse aortic constriction- induced cardiac hypertrophy in rats	21.9 and 87.6 mg/kg twice daily orally for 6 weeks	TETA (21.9 mg/kg/day): ↓ LVAW_d_ ↓ LVPW_d_ ↓ HW/TL ↓ cardiomyocyte size TETA (87.6 mg/kg/day): ↔ LVAW_d_ ↔ LVPW_d_ ↔ HW/BW ↔ cardiomyocyte size	[14]
Animal	STZ-induced diabetic cardiomyopathy in rats	20 mg/day in drinking water for 8 weeks (post-treatment)	↓ LV mass/BW	[51]
Animal	STZ-induced diabetic cardiomyopathy in rats	8–11 mg/day orally for 6 weeks	Improved myocardial structure	[80]
Animal	STZ-induced diabetic cardiomyopathy in rats	30 mg/day in drinking water for 7 weeks (preventive)	↔ HW/BW	[33]
Animal	STZ-induced diabetic cardiomyopathy in rats	20 mg/day in drinking water for 8 weeks (post-treatment)	↓ HW/BW	[34]
Human	Type 2 diabetic patients with LVH (*n* = 15)	600 mg twice daily orally for 12 months	↓ LVM_bsa_	[40]
Human	Patients with hypertrophic cardiomyopathy (*n* = 20)	300 mg twice daily orally, increased after 1 week to 600 mg daily if tolerated for 6 months	↔ LVM_bsa_	[23]

BW, body weight; HW, heart weight; LVAW_d_, diastolic left anterior ventricular wall thickness; LV, left ventricle; LVH, left ventricular hypertrophy; LVM_bsa_, left ventricular mass per body surface area; LVPW_d_, diastolic left posterior ventricular wall thickness; STZ, streptozotocin; TL, tibial length; ↓, significant decrease; ↔, no difference.

## Data Availability

Data sharing not applicable.
